# Strategies to reach, involve, and maintain underserved populations in health intervention research: a systematic review of reviews

**DOI:** 10.1186/s12939-026-02858-3

**Published:** 2026-05-07

**Authors:** Jeroen Pronk, Hristiyanna Ivanova, Zeena Harakeh, Annemarie Schuller, Pepijn van Empelen, Hilde van Keulen

**Affiliations:** 1 Expertise Group Child Health, The Netherlands Organization for Applied Scientific Research, Leiden, The Netherlands; 2 Department of Dentistry Orthodontics, Section Behavioral and Comtemplative Dentistry, University Medical Center Groningen, Groningen, The Netherlands; 3 Department of Health, Medical and Neuropsychology, Leiden University, Leiden, The Netherlands

**Keywords:** Underserved populations, Hard-to-reach populations, Inclusive research, Health interventions, Recruitment, Retention, Review

## Abstract

**Background:**

Certain population subgroups, including people with lower socioeconomic status, racial and/or ethnic minorities, and individuals with lower literacy skills, face greater health challenges and participate significantly less in health intervention research. This leads to non-generalizable findings and interventions that fail to meet their needs. Despite the recognition of barriers, such as mistrust, limited access, and communication challenges in previous research, strategies to overcome these barriers are still insufficiently implemented. This review of reviews aimed to synthesize existing intervention strategies for improving the recruitment and retention of underserved populations in health intervention research across health domains and assess evidence on their effectiveness.

**Methods:**

A systematic review of existing reviews was conducted in three databases (i.e., PubMed, PsycINFO, and Scopus) to identify, summarize, and synthesize strategies used to reach, involve, and maintain underserved populations in health intervention research. The following inclusion criteria were applied: (systematic) reviews and/or meta-analyses published in English between January 2013 and January 2026, with a focus on strategies for the inclusion of underserved populations in health intervention research.

**Results:**

A total of 18 reviews published between 2014 and 2026 met all inclusion criteria, the majority (*N* = 11) being systematic reviews. The findings revealed considerable overlap across reviews, underscoring that certain strategies are frequently used. A comprehensive summary of these strategies is provided, categorized based on their primary goal: (1) encouragement strategies to improve outreach to underserved populations, (2) facilitation strategies to improve their participation, and 3) retention strategies to maintain their participation. However, retention was rarely addressed as a primary focus in the included reviews.

**Conclusions:**

After integrating evidence across reviews from diverse health domains and underserved populations, the findings of this review of reviews highlight the importance of a multifaceted recruitment strategy that incorporates and/or combines approaches and serve goals throughout the health intervention research process. Researchers should: (1) tailor research materials, design and interaction methods, (2) collaborate with local organizations and healthcare providers, and (3) offer flexible participation options and incentives. Additionally, regular assessment and adaptation of the intervention materials are crucial to maintain participant engagement and improve the intervention’s effectiveness.

**Supplementary Information:**

The online version contains supplementary material available at 10.1186/s12939-026-02858-3.

Certain population subgroups experience disproportionate disease burden and reduced well-being [1–5]. These include subgroups of people with low socioeconomic status (SES), racial and ethnic minorities, elderly individuals, those living in rural areas, and/or those with lower literacy skills. Health disparities within these population subgroups are not only influenced by individual factors but also by micro-, meso-, and macro-level social and structural determinants of health (e.g., income inequality, educational opportunities, housing conditions, employment security, systematic discrimination; [6]). As a result, these people face increased health risks due to unhealthy lifestyle patterns, poor living conditions, and broader constraints that limit their access to proper resources and opportunities (e.g., limited access to education, unstable employment, unfavorable neighborhood environments; [7, 8]). Moreover, they are more likely to encounter financial, cultural and/or linguistic barriers (e.g., discriminatory policies, lack of transportation infrastructure) to accessing public health and healthcare services [9]. In this article, we refer to the population subgroups defined by these characteristics as underserved populations. Health benefits and equity can be achieved for underserved populations by making health interventions and other health promotion services—that is, all planned actions and/or strategies designed to improve health outcomes, prevent disease, or promote well-being at the individual, community, or population levels—more inclusive and accessible [9]. Inclusivity can, for example, be improved by respecting cultural, linguistic and socioeconomic diversity and making them more relevant and acceptable to all population subgroups. Accessibility, on the other hand, can be improved by reducing (practical) barriers to participation such as transportation costs and health literacy. Nevertheless, underserved populations generally participate less in and benefit less from research in general and health interventions specifically.

Insufficient inclusion of certain population subgroups is a frequently encountered issue in health intervention research (i.e., the systematic study of the design, implementation, effectiveness, and impact of health interventions to generate evidence to further practice and policy) [10–12]. The issue stems from researchers’ inability to properly recruit from, and maintain the involvement of, participants from all layers of society [13]. The exclusion of underserved populations causes skewed data collection and consequently results in non-generalizable conclusions [14, 15]. As a result, health interventions may well turn out to be less effective as they do not reach and/or align with the needs and desires of all critical target populations [16, 17]. Moreover, research and/or policy recommendations may well contribute to increasing health inequities when these are derived from research that inadvertently excluded (or underserved) certain target groups. For example, women have traditionally been underserved within cardiovascular research and clinical guidelines still insufficiently account for sex-specific symptoms and risk factors, which complicates accurate diagnosis and treatment of cardiovascular disease in women [18]. Similarly, health policies derived from studies in higher-income or majority populations often insufficiently address the cultural, linguistic and/or financial barriers faced by racial and ethnic minorities and/or low-SES populations, thereby increasing health inequities [9]. To overcome such critical issues, it is crucial that we gain more insight into strategies that researchers can implement to overcome structural barriers in reaching, involving and maintaining underserved populations in health intervention research.

Previous review studies have explored the reasons behind researchers’ difficulty in properly recruiting and retaining underserved populations in (health intervention) research [10, 11, 15, 19]. Important barriers include: (1) mistrust in research and researchers, particularly among ethnic minorities, (2) insufficient access, awareness and/or socioeconomic constraints (e.g., access to healthcare facilities, awareness of research studies and their benefits, inability to take off from work or come to testing locations), (3) problems in communication and sustained participation due to cultural or language differences and/or lower health literacy skills (e.g., misunderstandings or inability to understand study materials), and (4) researcher and institutional inadequacies, such as biases and stereotypes of researchers about underserved populations that influence recruitment and study design, and lack of infrastructure and resources within institutions to facilitate underserved populations to participate. Previous review studies have also examined strategies to overcome these (and other) barriers [10, 11, 20–23]. These strategies can be amongst others: (1) participant-oriented (e.g., providing participants with culturally and linguistically appropriate materials and communication, and offering them incentives and compensation for participation, (2) research(er)-oriented (e.g., using more flexible and accessible study designs, and training researchers and other staff to be more sensitive and respectful towards vulnerable population participants’ backgrounds and needs) and (3) recruitment-oriented (e.g., facilitating participation through stronger community engagement and partnerships).

As a result, previous reviews have clearly enriched our knowledge of problems and solutions with respect to the inclusion and sustained involvement of underserved populations in health intervention research. However, the generalizability of findings from existing health intervention research reviews [11, 12, 22–37] is hampered by: (1) a limited focus on specific subpopulations (e.g., ethnic minorities), specific behaviors or diseases (e.g., HIV/AIDS), and/or specific phases of the research process (e.g., recruitment only), (2) providing insight into only facilitators or barriers to (sustained) participation, rather than recruitment and/or retention strategies, and (3) not addressing or providing information about the effectiveness of recruitment and/or retention strategies. We aim to overcome these limitations by means of a systematic review of reviews and to provide a more rigorous and integrated view of strategies researchers can implement to improve the inclusion of underserved populations in health intervention research. Our synthesis offers a unique contribution to the literature by integrating evidence across reviews from diverse health domains and underserved populations. Examining such reviews collectively rather than in isolation, will help to identify cross-review patterns, areas of convergence and divergence, and persistent gaps in the literature. As such, we will be able to provide a more comprehensive and actionable understanding of strategies for successful recruitment and retention of underserved populations in health intervention research.

Specifically, in this review of reviews we will synthesize previously reviewed (effective) intervention strategies separately for critical phases of the research process in which issues with vulnerable populations are encountered, namely for: (1) reaching participants (i.e., gain better access to underserved populations), (2) involving participants (i.e., stimulate underserved populations to participate), and (3) maintaining participants (i.e., support retention of underserved populations). We will also explore whether sufficient evidence exists to inform on the (expected) effectiveness of including these strategies in health intervention research. Finally, it must be noted that participatory approaches, such as community-based participatory research and co-design, also offer valuable strategies for engaging underserved populations in health intervention research [38, 39]. While participatory approaches can indirectly support recruitment and retention, their mechanisms are typically not reported as recruitment and/or retention strategies directly. Therefore, such approaches fall outside of the scope of our review of reviews and were not explicitly included in our literature search and data extraction strategy. That is, our review of reviews focused on examining strategies directly relevant to recruiting and retaining underserved populations in health intervention research specifically.

## Methods

### Search strategy

We aimed to identify and synthesize the key findings of existing (systematic) reviews with respect to the strategies used to reach, involve, and maintain underserved populations in health intervention research. We used the updated Preferred Reporting Items for Systematic reviews and Meta-Analyses [40] methodology to execute a systematic review of reviews and report our findings. We executed a first literature search in PubMed in Q1 of 2023 as: (“Vulnerable Populations“[Mesh] OR “Minority Groups“[Mesh] OR “Health Disparate, Minority and Vulnerable Populations“[Mesh: NoExp] OR “hard-to-reach”[tiab] OR “difficult-to-reach”[ti] OR “hidden population*”[tiab] OR “unserved population*”[tiab] OR “disadvantaged population*”[tiab] OR “underrepresented population*”[tiab] OR “underrepresented groups”[tiab] OR “hidden populations”[tiab:~2] OR “invisible populations”[ti:~2] OR “vulnerable populations”[ti:~2] OR “marginalized populations”[ti:~2] OR “minority populations”[tiab] OR “minority communit*”[tiab] OR “respondent driven”[ti] OR “hard to contact”[tiab] OR “hard to engage”[tiab] OR “minority participation”[tiab] OR minorit*[ti] OR vulnerab*[ti] OR underrepresent*[ti] OR underserved[ti] OR hidden[ti] OR ethnic[ti] OR racial[ti] OR frail*[ti] OR migrant*[ti] OR refugee*[ti]) AND (“Patient Selection“[Mesh] OR “Sampling Studies“[Mesh] OR sampling[tiab] OR sampl*[ti] OR recruit*[ti] OR reach*[ti] OR (barrier*[ti] AND (contact*[ti] OR treat*[ti] OR care[ti] OR find*[ti])) OR (recruit*[tiab] AND strateg*[tiab]) OR (research[ti] AND engag*[ti]) OR ((disparit*[tiab] OR recruit*[tiab]) AND (“social media”[tiab] OR “digital health”[tiab] OR mhealth[tiab] OR smartphone[tiab]))) AND (“Meta-Analysis” [pt] OR “Systematic Review” [pt] OR “systematic review”[tiab:~2] OR “systematic overview”[tiab:~2] OR “scoping review”[tiab] OR “umbrella review”[tiab] OR “rapid review”[tiab] OR metaanal*[tiab] OR meta-anal*[tiab] OR systematic[sb]). This literature search was restricted to articles published from January 2013 onwards (i.e., approximately the last 10 years at the time of the first search). We executed a second literature search in January 2026 with two aims: (1) to extend the literature search in PubMed to January 2026, and (2) to expand the full search—with comparable search terms—to Scopus and PsycINFO.

### Inclusion/exclusion criteria, review selection, and data extraction

The inclusion criteria for articles in this systematic review of reviews were as follows: (1) type of article is review (e.g., meta-analyses, systematic review, scoping review, or rapid review), (2) the article focuses on strategies to reach, involve, and/or maintain underserved populations in health intervention research, (3) the study is published in English in an academic peer-reviewed journal, and (4) study was published within the last 10 years at the time of the—first—search (i.e., from January 2013 onwards). Articles were excluded if these criteria were not met and/or when the article itself was some type of review of reviews.

The screening process for the selection of the reviews consisted of two steps: (1) title and abstract screening, and (2) full-text screening and review. During both steps, the articles were equally divided among four authors (AS, HVK, JP, and ZH) and independently screened in sets of two authors (HVK-JP and AS-ZH). Disagreements were resolved through consensus discussion plus additional review—where needed—during meetings between these four authors and PVE. Finally, data were extracted by three authors for the final set of review articles (HI, HVK, and JP). During this phase, the reference lists of the included articles were also scanned to identify potential review articles not identified through the initial search. The following data were extracted: (1) type of review, (2) objectives, (3) number of studies included, (4) inclusion/exclusion criteria, (5) study population characteristics, (6) type of strategies used for reaching, involving, and maintaining the study population (including outcomes), and (7) where available, information regarding the effectiveness of these strategies. These data were used to report on (1) the strategies used to reach, involve, and maintain underserved populations in health intervention research, and (2) where possible, also the effectiveness of these strategies.

### Quality assessment of reviews

The quality of the health intervention review articles included in this systematic review of reviews was assessed via de Vet et al.’s quality assessment rating scale [41]. This rating scale consists of 8 items (see the left column of Table 1 for a description of all items) that are scored as 0 (no) or 1 (yes). These items are summed to obtain a quality assessment rating ranging from 0 to 8, with quality ratings of 0–4 labelled weak and quality ratings of 5–8 labelled strong.

**Table 1:** Quality assessment of included reviews based on de Vet et al.’s (2011) rating scale [41]

Item:	[11]	[12]	[22]	[23]	[24]	[25]	[26]	[27]	[28]	[29]	[30]	[31]	[32]	[33]	[34]	[35]	[36]	[37]
**1.**	**Was there a clearly defined search strategy?**																	
	no	yes	yes	yes	no	yes	yes	yes	yes	yes	yes	yes	yes	yes	yes	yes	yes	yes
**2.**	Was the search strategy comprehensive? *																	
	no	yes	yes	yes	no	yes	no	yes	no	no	no	yes	no	no	no	no	no	no
**3.**	**Are inclusion/ exclusion criteria clearly stated?**																	
	yes	yes	yes	yes	yes	yes	yes	yes	yes	yes	yes	yes	yes	yes	yes	yes	yes	yes
**4.**	**Are the designs and number of included studies clearly stated?**																	
	yes	no	yes	yes	yes	yes	yes	yes	yes	yes	yes	yes	yes	yes	yes	yes	yes	yes
**5.**	**Has the quality of primary studies been assessed?**																	
	no	no	no	no	no	no	no	yes	no	yes	yes	no	yes	no	yes	yes	no	no
**6.**	**Did the quality assessment include study design**,** study sample**,** outcome measures**, or follow-up? **																	
	no	no	no	no	no	no	no	no	no	yes	no	no	yes	no	yes	no	no	no
**7.**	**Does the review integrate findings beyond describing or listing findings of primary studies?**																	
	yes	yes	no	yes	no	yes	yes	yes	yes	yes	yes	yes	yes	yes	yes	yes	yes	yes
**8.**	**Has more than one author been involved in the data abstraction process?**																	
	yes	yes	yes	yes	yes	no	no	yes	yes	yes	yes	yes	yes	yes	yes	yes	no	yes
Sum quality score***																		
	4	5	5	6	3	5	4	7	5	7	6	6	7	5	7	6	4	5
	weak	strong	strong	strong	weak	strong	weak	strong	strong	strong	strong	strong	strong	strong	strong	strong	weak	strong
**No. of included studies**																		
	21	68	21	41	5	12	47	28	31	9	7	14	58	43	25	43	37	7

Note. *A search is rated as comprehensive if at least two databases and the reference lists of examined papers were searched. **At least 2 out of 4 criteria are met. ***Sum quality score: weak (0–4), strong (5–8)

## Results

### Summary of included reviews

**Fig. 1:**
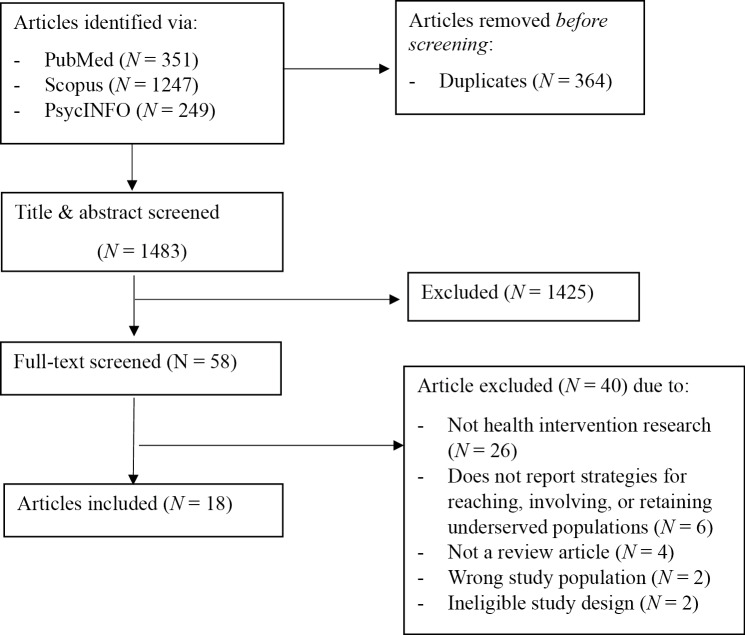
Flow diagram for the selection of included reviews

A total of 1847 articles were identified across databases (351 from PubMed, 1247 from Scopus, and 249 from PsycINFO). After removal of 364 duplicates, 1483 articles were screened independently by two authors to determine eligibility for inclusion in the systematic review of reviews. Screening was done in two steps (see Fig. 1 for the flow diagram): (1) title and abstract screening (*N* = 1483), after which 1419 articles were excluded; and (2) full-text screening and review of 64 articles. Disagreements between reviewers were resolved through inter-author discussion. After the screening process, 18 reviews remained that met all in-/exclusion criteria. Reasons for exclusion at the full-text screening stage are summarized in Fig. 1 and documented in more details in Supplementary Table S1.

A detailed overview of the included reviews is provided in Table 2. The 18 included reviews were published from 2014 to 2025 and consisted primarily of systematic reviews (*N* = 12), alongside scoping reviews (*N* = 4) and narrative or general literature reviews (*N* = 2). The number of included studies within the reviews varied greatly from 5 to 68. Most reviews focused on populations at risk for, or living with, specific health conditions (*N* = 12), including HIV, cancer, type 2 diabetes, mental health disorders, obesity, arthritis, and fibromyalgia. More than half of the reviews specifically targeted ethnic or racial minority groups (*N* = 10), such as British South Asians, Hispanic/Latinx (older) adults, Black and Indigenous adults, or minority ethnic groups more broadly. Other reviews addressed underserved or hard-to-reach populations in a more general sense (e.g., individuals with low SES, migrants, rural communities). All reviews described recruitment strategies aimed at reaching and involving underserved populations in health intervention research. Only one-third of the reviews (*N* = 6) also reported retention strategies or factors affecting sustained participation of underserved populations in health intervention research. Although community engagement and partnerships were frequently described as recruitment strategies, none of the included reviews explicitly focused on community-based participatory research or co-design as a central objective.

**Table 2:** Overview of included reviews

Reference	First author	Year	Type of review	Objectives	Number of included studies	Databases searched	Characteristics of the study population	Search timeframe
[11]	Heller	2014	Systematic review	Identify successful community-engaged interventions that included healthcare providers as a key strategy in addressing barriers to clinical trial enrollment of underrepresented patients.	21	Ovid Medline, EBSCO Megafile, EBSCO CINAHL	Underrepresented patients (e.g., ethnic groups, minorities)	January 2001 - October 2013
[12]	Machalicek	2022	Systematic narrative literature review	Report parent-mediated intervention research of children with or at risk for mental health disorders and to identify potentially effective recruitment and retention strategies for diverse participants in parent-mediated intervention research for children with autism.	68	PsychNET, MEDLINE	US child and parent (combination) populations with child mental health disorder (or at-risk; main focus on Autism Spectrum Disorder), focusing on minority populations (ethnicity and SES)	2011–2020
[22]	Masood	2019	Systematic review	Describe strategies for recruiting ethnic minority participants to UK clinical trials.	21	Medline, Embase, PsycINFO, CINAHL	All non-White English (UK) populations of 18 years and older (ethnic minorities)	Studies to April 2018
[23]	Pellegrini	2023	Scoping review	Identify strategies used to recruit and retain underrepresented populations and populations with arthritis or fibromyalgia into behavioral programs targeting exercise, physical activity, or chronic disease self-management.	41	Web of Science, Ovid Medline, CINAHL, PsycINFO, Cochrane	Individuals with arthritis, fibromyalgia, or underrepresented populations (incl. racial and ethnic minorities, rural residence, low socioeconomic status, or individuals with arthritis or fibromyalgia)	January 2000 - May 2022
[24]	Waheed	2015	Systematic review	Share strategies used by the research group in recruiting British South Asians and thereby encouraging other researchers to consider ethnic minority inclusion into research. Find strategies to overcome previously published barriers to recruitment of ethnic minority participants into mental health research by drawing from recruitment strategies used in research (RCTs of psychosocial interventions for depression) by the research group among British South Asians.	5	RCTs of psychosocial interventions for depression the University of Manchester research group has been involved with (SITARA, AMP, COINCIDE, ROSHNI-D)	British South Asian populations (primarily Pakistani)	Until 2019
[25]	Jones	2016	Literature review	Provide a general review of the use of social networking sites in the context of primary prevention of HIV. The article describes how social networking sites have been used in the recruitment of at risk individuals for prevention interventions, evaluation of the impact and reach of social networking sites campaigns, and the efficacy of social media approaches to deliver HIV-related information on sexual health outcomes.	12	PubMed, PsycINFO, ScienceDirect, Google Scholar, Georgia State University Library Database	Individuals at risk for HIV	2012 - July 2015
[26]	Sokol	2016	Systematic review	Assess reach and effectiveness of peer support among hardly reached individuals and peer support strategies used	47	PubMed	Those who are hardly reached (hard to reach, hard to locate, difficult to engage)	January 2000 to January 2015
[27]	Yang	2020	Systematic review & meta-analysis	Assess the efficacy of social network strategy used by HIV high-risk populations.	28	PubMed, Web of Science, Embase, Cochrane Library, ScienceDirect, Wiley	HIV high-risk populations including men who have sex with men, drug users, sex workers, bisexual or transgender persons and the populations who were at high-risk or lived in high-risk areas (data of the Centers for Disease Control and Prevention).	January 1999 - May 2019
[28]	Rodriguez	2023	Scoping review	Identify factors hindering/facilitating recruitment of Hispanic/Latinx American older adults (≥ 65 years) into clinical trials in the US.	31	PubMed, EMBASE (via Ovid)	Studies aiming to recruit or recruiting Hispanic/Latinx Americans aged ≥ 65; mixed disease areas (often cancer).	Searches conducted March 2022; included articles since inception of each database.
[29]	Nouvini	2022	Systematic review	Identify and characterize interventions that improve actual accrual of racial/ethnic minority adult cancer patients into cancer clinical trials.	9	PubMed, Cochrane CENTRAL, PsycINFO (Ovid)	Adult cancer patients from REM groups (Black, Hispanic, Asian, American Indian, Native Hawaiian/other Pacific Islander).	1993- May 2020
[30]	Nolan	2021	Systematic review	Examine the use of video education interventions to increase clinical trial participation among Black, Indigenous, and people of color adult cancer survivors.	7 (describing 6 distinct interventions)	Web of Science, Embase, PubMed, Cochrane Library, PsycINFO, CINAHL	Adult cancer survivors identifying as Black, Indigenous, and people of color; primarily Black and Hispanic survivors; oncology populations in the United States.	No date restrictions applied
[31]	Mahmood	2017	Narrative review	Characterize recruitment and retention rates of South Asian populations in behavioral interventions for type 2 diabetes and identify strategies used.	14	PubMed, CINAHL, EBSCO, PsycINFO	South Asian adults (Indian, Pakistani, Bangladeshi, Sri Lankan, Nepalese, Surinamese South Asian) living in Western countries; with type 2 diabetes or at risk.	1975 - October 2016
[32]	Isaacs	2016	Systematic review	(1) Examine reporting practices and prevalence of ethnic minority inclusion in telehealth RCTs for type 2 diabetes; (2) assess use of English language proficiency as an eligibility criterion and how it is operationalized	58 (reported across 79 articles)	MEDLINE, PsycINFO, EMBASE, CINAHL, CENTRAL	Adults (≥ 18 years) with type 2 diabetes, recruited in Western majority English-speaking countries (USA, UK, Canada, Australia, New Zealand, Ireland)	January 2000 - July 2015
[33]	Cui	2015	Review of clinical trials registration database and literature review	Examine recruitment and retention strategies (and rates) in obesity prevention/treatment trials targeting minority or low-income children with an obesity-related behavioral component.	43	ClinicalTrials.gov registry (CTRD), PubMed (specific for identified trials)	Minority or low-income children/adolescents aged 2–17 in completed home-, community-, and school-based obesity-related intervention trials.	CTRD search conducted on 6 March 2014
[34]	Wali	2024	Systematic review	Measure recruitment and retention rates and identify barriers and facilitators to recruitment and retention of ethnic minorities and migrants in community-based obesity prevention RCTs in Organization for Economic Cooperation and Development (OECD) countries.	25	CINAHL, Cochrane, Embase, Medline, PsycINFO, plus Google and Google Scholar	Ethnic minority, migrant, refugee, and Indigenous populations (children, adolescents, and adults) participating in community-based obesity prevention RCTs in OECD countries.	January 2000 - March 2022
[35]	Alarcón Garavito	2025	Systematic review	Identify factors that hinder and facilitate participation of minority ethnic groups in clinical trials.	43	Web of Science; PubMed; CINAHL Plus; Cochrane Library / CENTRAL	Individuals from minority ethnic groups.	January 2017 – October 2022
[36]	Pottinger	2025	Scoping review	Examine interventions that increase minority enrollment and retention in cancer-related clinical trials across the cancer care continuity.	37	PubMed, EMBASE, Scopus	Adult participants from underrepresented racial and ethnic minority groups (predominantly Black, Hispanic/Latino, Asian, Indigenous populations) involved in cancer prevention, screening, or treatment trials, mainly in the United States.	EMBASE: 2012 – March 2023
[37]	Biggs	2024	Scoping review	Identify, report, and evaluate effectiveness of interventions to improve representation of under-served groups in RCTs in the UK and Ireland.	7	PubMed	Underserved groups targeted: ethnic minority groups, socioeconomically disadvantaged, older people, and those with impaired capacity to consent, in UK/Ireland RCT contexts.	2000 – November 2021

### Quality of reviews

Table 1 describes the quality assessment of the 18 included reviews. Overall, the quality of 14 of the 18 reviews was strong. All reviews clearly stated their in-/exclusion criteria. In addition, 16 reviews clearly reported the design and number of included studies, and integrated findings beyond merely describing or listing findings from primary studies. Moreover, 15 reviews involved more than one author in the data abstraction process. Furthermore, while 16 of the 18 reviews reported a clearly defined search strategy, only 6 reviews reported a comprehensive search strategy (i.e., searching at least two databases and performing a check on reference lists of included reviews). Finally, only 6 reviews assessed the quality of included primary studies and only 3 of these included at least two of the following elements in their quality assessment: study design, study sample, outcome measures, and/or follow-up.

### Strategies to better reach, involve and maintain underserved populations

The results reveal significant overlap among the reviews, highlighting the widespread use of certain strategies across health domains. These strategies can be summarized into one or more of the three overarching goals: (1) Encouragement strategies to better reach underserved populations: These strategies focus on motivating and inspiring potential participants to take part in health intervention research. (2) Facilitation strategies to better involve underserved populations: These strategies focus on making the participation process easier and more accessible. (3) Retention strategies to better maintain underserved populations: These strategies focus on stimulating and supporting participants to continue their participation throughout the research process. Some of the strategies described can serve more than one of these goals, making the distinction between them not always clear-cut. Below, we classify the strategies according to their primary goal category. We did not identify any strategies exclusively aimed at retention; therefore, encouragement and facilitation strategies are described separately, with an indication of those that also serve retention goals. Table 3 provides a comprehensive summary of all strategies identified, which are presented independently to reflect their distinct roles.

**Table 3:** Overview of strategies to better reach, involve and maintain underserved populations in health intervention research

Encouragement strategies to better reach underserved populations
** Engaging target populations**
Partnership with community members, leaders, organizations prior to protocol development, study start or recruitment strategies, or after the study start and make modifications to the protocol and implementation plan based on feedback if enrollment targets are not met. Use community members or community coordinators to recruit participants [11, 29, 31, 34, 36, 37]
Engage with community and family – support for project via religious leaders/setting, community-based organizations for recruitment and referrals, self-referrals from participants in family/friend circles [22, 28–31, 36]
Engage with community at specific for the target group public events, to increase visibility [25, 28, 34, 36, 37]
Involve known public figures (e.g., politicians, artists, athletes), ethnic celebrities [31] or local leaders [34] who stand behind the health appeal/ intervention [24] Hiring research staff from the community or representatives (community/cultural demographics). There is a key role for research nurses and coordinators to identify potential participants and ask them to participate [11, 29, 31, 34, 36, 37]
Organize informational sessions and presentations at community specific locations (churches and synagogues, retirement or assisted living communities, civic clubs, community agencies or organizations, and clinics) [23, 35]
Involving stakeholders in the development of the intervention [12, 30, 34]
Involving participants in care (e.g., patients nominate peers to become supporters) [26, 34, 35]
Empowerment – engaging participants to be active members of the health team [26]
Frequent contact – regularly engagement with participants with minimal every 2 weeks during the intervention period [26]
Assertive contact – contact initiated/maintained by peer supporters instead of 1 initial contact and waiting for participants to ask for assistance [26]
Monitored contact – supporters documented interactions with participants to monitor progress and improve record keeping [26]
** Stimulating collaboration with end users and/or intermediaries**
Training and educational programs to inform communities about the nature of clinical trials and address mistrust of the research process, including training people who work to increase enrollment generally [11, 34, 36] Educate provider staff about clinical trial methodology and research practices needed to improve understanding and supportiveness of the trial (especially in double-blinded study) [11, 29] Culturally specific recruitment training for staff [22, 29]
Implicit support – just being there (e.g., meeting or coffee to talk) [26]
Ready availability of supporters – participants were able to contact supporters when needed [26]
Fund a position for a community member, who acts as a link towards the community and builds engagement [24] Face-to-face meetings with the principal investigator, community research staff or coordinators with healthcare providers before the start of the study to explain the study, address concerns, gain commitment in assisting with recruitment and enrollment. Engaging community providers means prior relationship building with the principal researcher or expected provider association to make contact, this is successful [11, 37]. Not successful are cold calling, letters and faxing information [11]
Recruitment and referral via community organizations such as schools, direct referral, community agencies or settings through outreach, physicians or service providers, university clinic or faculty, trusted persons, child protective services [12, 28, 31, 35]
Community partnerships – collaborate with other organizations to disseminate information, ensure awareness of program, acknowledge/overcome service limitations via referrals, e.g., peer supporters as liaisons to community resources and healthcare system and made referrals to providers when needed [26, 36, 37].
Collaborating with media – use specific for the target group media (TV, newspaper, radio) to spread awareness about the intervention [24, 28, 31]
** Tailoring recruitment, research materials and communication**
Use materials and direct outreach with culturally sensitive content (e.g., narratives from culturally/ethnically similar individuals) and style and in the native language [11, 29, 30, 32–34, 36]. Adapt framing and terminology of interventions to align with cultural or religious norms of the target group [31] Use plain language and easy-to-read recruitment materials [28, 30–32, 34, 36, 37] or tailor materials to the proficiency level of target group (e.g., face-to-face instead vs. written invitations, more graphics than words) [28, 31, 32] Use interpreters / dedicated language support when language barriers exist [28, 32, 34, 35]
Use topic specific social media pages and/or campaigns (e.g., Facebook advertisements), containing topic and/ or target group specific language, photos (including people tagging) and videos [25]
Adaptation of screening and outcome measures – translation, adaptation, explanation [22] Avoid subjective or non‑standardized language proficiency screening to prevent exclusion of minority participants [32] Improve completeness/accuracy of race–ethnicity fields in (electronic) health records to enable identification of eligible minority participants [35]
Study invitation process – use multilingual recruitment materials [22] Train research staff in empathetic communication [29], the use non-stigmatizing words, definitions and explanations about health topics (e.g., inviting people for a “screening” is more widely accepted than inviting people for a “mental health assessment”) [24] and explaining clinical trials [28]
Intervention administered through a bilingual interventionist [12, 28, 31, 32]
Translating materials [12, 28, 32]
Tailored content – supporters recognized needs and tailored interventions to needs [26] Widen inclusion criteria to reduce systematic exclusion of underserved groups [28, 31, 37] Highlight eligibility criteria in recruitment materials [37] Highlight how the study will benefit specific communities in the recruitment communication/materials (community benefit framing) [32, 34, 35] Use targeted email distribution lists, if appropriate for the target population [23] Use legally and ethically approved data sources or trial‑matching systems to identify potentially eligible participants prior to recruitment outreach [28] Recruitment via flyers or mailings, word-of-mouth, radio or newspaper, online or social media, workshops or open house, self-referral (families contacting researchers) [12, 28, 29, 33, 37] Use multiple communication channels simultaneously (e.g., flyers, radio, text messages, phone calls, emails, letters, reminders) [34] Recruitment via community through peer-led social networking sites program – recruitment of community members initiated by peer leaders, following by the community members recruiting their friends and connections through social networking sites [25] Recruitment via social networking sites (e.g., Facebook) with attractive banners or advertisements – effective for recruitment of young, urban population from a large geographical area (large reach) [25] Participant response cards (patients interested could call a toll-free number), phone line [28, 34], or research staff being present in the office of the healthcare provider to create awareness of the trial among providers and patients ([11] Sending letters of support or invitations to patients, approved and signed by the community providers to increase awareness of the study, including follow-up with them by the study staff [11] Pilot-testing of recruitment approaches prior to the start of the study, or modify recruitment methods and sites when enrollment targets are not met; e.g., staggered intervention-start date with rolling enrollment of practices and participants over 9 months, with added strategies such as asking interesting individuals (who signed and interest card) to refer participants from their own social networks, providing community recruiters with incentives (5 dollar gift card/referral enrolled) to recruit participants from their social networks [11]
** Building trust and respect**
Being non-judgmental [26]
Relate to and empower people [26] Proactively address anxiety/ provide comfort to create a safe, friendly recruitment experience [28]
Have similar characteristics as target population (cultural, demographic, socioeconomic) [26, 32]
Provide a contact person (e.g., senior research member) with similar characteristics to the target population (e.g., speaking the native language) [24]
Co-organize local events, related to cultural and religious festivities, relevant to the community [24]
Avoid contacting people on special for the target group days (e.g., religious festivities and rituals) [24]
Participant confidentiality – avoid the use of research staff, who resides within the same community as the participants to decrease participant hesitancy to disclose sensitive information [24]
Reassure participants with a particular profile (e.g., immigration status, benefits entitlement) that their information will not be shared with institutions (e.g., immigration authorities) [24] Provide culturally specific food/ drinks to show respect and gain trust [35]
** Passive inclusion**
Passive exclusion makes enrollment faster: with active enrollment, providers have to agree to include patients in the study. With passive enrollment, providers notify research staff if they want to exclude a patient within 10 working days after notification of the patient eligibility status [11, 37]
**Facilitation strategies to better involve underserved populations**
** Increasing accessibility**
Engaging providers, hospitals, and/or participants in the underserved communities by recruiting local, community practices caring for underrepresented populations [11]
Conduct regular (e.g., weekly) outreach visits by navigators to community care sites (e.g., community hospital) to support recruitment [29] Combine outreach with community-service elements (e.g., offering free health checks) to improve face-to-face recruitment [28]
Conduct trials in the community instead of at an academic setting to create more opportunities for trial enrollment [11, 32, 34, 36, 37]
Training key members of a community to become peer-leaders, who then deliver the intervention within their social network. The community members act as an access point to underserved populations [27]
Transportation assistance or reimbursement [11, 29, 34, 36, 37]
Recruitment sites – recruit in ethnically dense areas [22, 28]
Offer flexible services responding to needs of participants (e.g., choice of contact method) [26, 37]
Offer alternative ways of participating (e.g., hybrid, fully online, streaming on mobile devices, phone-based intervention (e.g., mobile apps), individual sessions) [24, 25, 32, 35, 37]
Listen to feedback [26]
Offer flexible opening hours (e.g., evenings/weekend) [26, 28, 34] Provide devices or equipment required for participation in the intervention (e.g., mobile phones, glucose meters) to reduce participant financial and practical burden [32]
** Incentives**
Provide incentives to participants and/or providers (e.g., education materials or monetary grants) for recruitment and/or reimbursement for recruitment and study expenses, such as, monthly stipend to the care provider for high referral rates [11, 37]. Mention those in invitation letters or during the study recruitment [37] Offer devices or equipment as a participation benefit (e.g., self‑monitoring devices or mobile phones) [32]
Organize end of intervention ceremony and/ or provide certificates for completion to participants [24] Providing services wanted, such as, resources to address financial or housing issues when facing these [26]
**Retention strategies to better maintain underserved populations**
** Tailoring research (design), materials and communication**
Researcher and participant communication – linguistic matching or translation services, ethnic matching, gender matching, awareness of cultural practices and norms [22, 30, 34, 36, 37] Translation of information sheets [22, 34] and education materials [12, 34] Provide assistance to participants with literacy or form‑completion difficulties [36, 37]
Use of culturally relevant themes within the intervention [12, 33, 34] Adapt intervention to priority population [23, 33, 34, 37] Use a delayed or alternative intervention for the control group [33] Continuity of a dedicated patient navigator or research staff member throughout the study to support retention [28, 35, 36] Ongoing cultural‑competency training and reflective debriefing for research staff to support sustained engagement [28, 34, 35]
** Increasing accessibility**
Flexible scheduling [12, 28, 29, 31, 33, 34, 37]
Frequent appointment reminders [29, 33, 34, 37] and emphasis on importance of study visits [23] Collect complete contact information at baseline and maintain a participant tracking database to facilitate participant tracking [33]
Choice of interview/testing location [22, 33, 34]
Choice of intervention [12] Offer make-up sessions for missed intervention sessions [29, 31, 33, 37] Transportation assistance or reimbursement [29, 31, 33, 34, 36] Providing research staff (e.g., nurses and coordinators) on location and organizing a follow-up with them and patients identified by providers [11, 28, 33, 35, 36] Arrange regular meetings between researchers, community practice providers and staff to address study issues [11, 35] Maintain engagement through newsletters, social gatherings, or community events during the intervention [28, 34, 36] Send personalized letters to participants who are difficult to reach to schedule data collection appointments [33, 35] Support for supporters – engagement through debriefing sessions and opportunities for supporters to share challenges [26] Follow-up through home visits or phone calls [22, 31, 34], reminders [12, 31], audio-recorded information transmissions [22] or texts [31, 34] Ensure personalized and consistent follow-up contact with participants, such as thank-you notes, postcards, or project newsletters [28, 33–37]
** Incentives**
Money, (grocery) gift cards [12, 31–34], gifts, food, recipe books, exercise equipment [33], transportation cards, recognition certificates [31]
Monetary and non-monetary incentives [23, 36, 37] Free services, child care or toys [12, 31, 33, 34] Offering food and / or drinks [12, 33]

#### Encouragement strategies to better reach underserved populations

Strategies found that are aimed at encouraging underserved populations to participate in health intervention research are directed at the target population itself (end-users), whereas others specifically target community healthcare providers, organizations and facilitators (intermediary).

##### Engaging target populations

 Direct and indirect interactions with the target population (also referred to as the “community”) are crucial for healthcare intervention promotion. Engaging community members and stakeholders is highly recommended prior to recruitment to develop suitable study protocols, and after recruitment to modify the protocol and implement feedback [11, 12, 29–31, 34, 36, 37]. Contact can for example be initiated during public events specifically organized for the target population (e.g., religious or cultural festivities) [25, 28, 34, 36, 37]. Involving respected local leaders or well‑known public figures from the target community who endorse the intervention can further increase awareness, credibility, and community trust [24, 31, 34]. Co-organizing local events and holding informational sessions at community-specific locations (e.g., churches, retirement communities, civic clubs, and clinics) can further enhance intervention visibility and community engagement [23, 24, 35].

Maintaining regular interactions with participants and initiating follow-up instead of waiting for participants to reach out, are not only effective as engagement strategies but also towards the retention of underserved populations [23, 26, 29]. Monitoring and documenting interactions with participants helps to track progress and ensure continuous engagement [26].

###### Stimulating collaboration with end users

Community members or coordinators can aid with recruitment efforts, including encouraging participants to refer members of their family and friend networks [11, 22, 28–31, 36]. Training community members responsible for recruitment is vital in addressing mistrust and informing about the importance of clinical trials [11, 23, 29, 34, 36].

###### Stimulating collaboration with intermediaries

Partnering with other organizations, such as healthcare providers, schools, and community agencies, can overcome service limitations through participant referrals and connections to community resources and healthcare systems [12, 26, 36, 37]. Peer support provided by community health workers in the form of emotional, social and practical assistance is proven effective in various trials as both an encouragement as well as a retention strategy [26, 36, 37]. For example, participant navigators, such as community nurses who screen and engage with community members, have proven effective in identifying participants from underserved populations [11, 29, 31, 34, 36, 37]. Similarly, establishing a community-based infrastructure that supports participant inclusion by implementing eligibility screening programs within public hospitals will further aid this process.

Furthermore, organizing regular face-to-face meetings between healthcare providers and the principal investigator or research staff to address concerns, study-related issues and clarify commitments regarding recruitment aid, fosters a successful partnership [11, 26, 37]. An essential component of the research partnership is making sure healthcare providers are trained in reaching underserved populations and building trust [23, 29]. This also includes, for example, training them in clinical trial methodology [11, 29] and culturally specific recruitment practices to ensure proper practices [22, 29]. Finally, hiring representative research staff from the community [11, 29, 31, 34, 36, 37] and appointing key community members [24] to deliver the intervention within their social network are effective strategies for accessing underserved populations [26, 27].

##### Tailoring recruitment, research (design), materials and communication

 Tailoring strategies that are used for recruitment to the target population (e.g., communication resources, delivery mode, use of culturally sensitive content and language) ensures more success in the recruitment of underserved populations in healthcare interventions. A range of cost‑effective communication channels can be used due to their high recruitment potential, including flyers, direct mail, targeted email distribution lists, letters of support, word-of-mouth, and social media [11, 12, 23, 25, 28, 29, 33, 37]. Attractive banners and advertisements are thereby essential to elicit participation interests. In addition, participant-initiated methods, such as a response card with a toll-free number to express interest, are another example of a successful recruitment resource [11, 28, 34].

Social networking platforms (e.g., Facebook) are specifically suitable for reaching young, urban populations from a large geographical area [25]. (Peer-led) social networking strategy programs, which are based on linking people from underserved populations with similar characteristics are effective recruitment and retention strategies [25, 27]. Furthermore, community-specific media channels, such as TV, radio and newspapers are seen as effective for reaching a broad population and increasing awareness [23, 24, 28, 31].

Beyond channel selection, adapting materials to be culturally sensitive in content, style, and language, and providing translations, ensures accessibility and relatability for diverse populations [11, 12, 22, 28–30, 32–34, 36]. This holds true throughout the entire intervention research process—from study invitation, to screening and measuring outcomes [22, 25]. When recruiting via social media pages or campaigns, using language that resonates with the target demographic and incorporating relevant photos/videos (including tagged individuals) improve engagement [25]. Using plain‑language and easy‑to‑read recruitment materials (e.g., more visuals), or tailoring materials to the literacy and language proficiency level of the target group (e.g., face‑to‑face invitations instead of written materials), further supports accessibility [28, 30–32, 34, 36, 37]. This strategy also contributes to retention by reducing misunderstanding and burden. To overcome language barriers, it is recommended to use interpreters, bilingual interventionists or dedicated language support [12, 28, 31, 32, 34, 35].

Towards retention, providing assistance to participants with literacy or form‑completion difficulties helps prevent drop‑out during follow‑up measurements and administrative procedures [36, 37]. Moreover, collecting full contact information at baseline and maintaining a participant tracking database facilitates follow‑up and retention [33]. Sending personalized letters to difficult to reach participants also supports retention [33, 35], as well as ensuring personalized and consistent follow‑up contact, such as thank‑you notes, postcards, or project newsletters [28, 33–37].

Tailoring is also relevant to the process of recruitment and study design. Pilot-testing recruitment strategies before the beginning of a study allow for the refinement of approaches and protocols based on real-world feedback [11]. To mitigate negative perceptions related to clinical trials, it is important to ensure research staff uses non-stigmatizing language, definitions, and explanations about health topics [24, 28, 29]. For example, inviting people for a “screening” is generally more acceptable than inviting them for a “mental health assessment”. For retention, using a delayed or alternative control-group intervention design reduces attrition by maintaining control-group participants’ motivation [33].

Additional tailoring strategies that can be used are widening the inclusion criteria to reduce systematic exclusion of certain underserved groups [28, 31, 37], highlighting eligibility criteria in recruitment materials [37], avoiding language‑proficiency screening to prevent exclusion of underserved populations [32], and highlighting how study participation may benefit specific communities (community‑benefit framing) [32, 34, 35].

Finally, the translation of intervention materials and information also serves as a retention strategy, as participants’ full awareness is needed not only to obtain their consent but also during any follow-up moment in the research process [12, 22, 28, 32]. Moreover, adapting interventions to priority populations ensures relevance for these populations and improves their ongoing participation [31]. This also includes the use of follow-up methods that are appropriate for the target population, such as home visits, phone calls, motivational interviewing, and reminders [12, 22, 23].

##### Building trust and respect

 Building trust, being non-judgmental, and showing respect are key factors for a successful engagement with the community as well as for retention of participants [26]. Employing research staff and contacts who share similar cultural, gender, linguistic, and socioeconomic characteristics with the target population fosters trust, relatability and facilitates communication [22, 24, 26, 32]. Moreover, providing a central contact person (e.g., senior researcher) with similar characteristics to the target population, also strengthens trust and accessibility [24]. When these individuals ensure confidentiality and reassure participants that their information would not be shared with others, particularly with regards to sensitive information (e.g., migration status), their concerns are likely alleviated [24]. In extension, proactively alleviating participants’ anxiety and providing comfort, friendliness, and safety, support trust‑building during recruitment [28].

Moreover, avoid scheduling appointments on significant religious or cultural days shows respect for participants’ beliefs and practices [24]. Co‑organizing local events with community-relevant festivities can further support trust‑building and respectful engagement [24]. Providing culturally specific foods or drinks as a sign of respect can also build trust [35].

Towards retention, providing continuity via a dedicated patient navigator or research staff member throughout the study helps build trustworthy relationships for the long‑term [28, 35, 36]. Moreover, continued cultural‑competency training and reflective debriefing for research staff helps to prevent participant disengagement over time [28, 34, 35]. Participant engagement via newsletters, social gatherings, or community events during the research period helps to reinforce participants’ connection to the study and community [28, 34, 36].

##### Passive inclusion

 Passive enrollment is preferred over active enrollment. The former speeds up the recruitment process by allowing healthcare providers to decide if they want to exclude patients within a set time, compared to active enrollment, which requires them to actively agree to include patients in the study [11, 37].

#### Facilitation strategies to better involve underserved populations

In addition, strategies were directed at either facilitating participation opportunities for underserved populations or reducing barriers to their participation. These factors include flexibility of the location and delivery mode of the intervention, and the provision of incentives for participation.

##### Increased accessibility

 Conducting healthcare interventions within the community, as opposed to traditional academic settings, creates more opportunities for participation, greater accessibility, and reduces the enrollment barriers [11, 32, 34, 36, 37]. For example, it is recommended to establish recruitment sites in ethnically dense areas [22, 28]. Other services that facilitate participation and support retention by preventing drop-out include providing transportation [11, 29, 33, 34, 36, 37], offering flexible intervention options (e.g., hybrid sessions, streaming on mobile devices, phone-based interventions and individual sessions) [24, 25, 32, 35, 37] and allowing participants to choose their preferred method of contact [26, 37]. Maintaining flexible hours and scheduling of appointments [12, 26, 28, 29, 31, 33, 34, 37], and providing devices or equipment required for participation in the intervention (e.g., mobile phones or glucose meters) can further reduce practical barriers [32].

Other strategies to support accessibility include regular outreach visits to community care sites [29] and combining outreach with community‑service (e.g., free health checks) to lower participation barriers [28]. Actively listening to participant feedback and adapting procedures accordingly, also improve accessibility more broadly [26, 27]. Towards retention, offering make‑up sessions for missed research sessions will allow participants to remain engaged despite for example competing demands [29, 31, 33, 37].

##### Incentives

 Offering free services (e.g., childcare, toys) [12, 31, 33, 34], (non-)financial incentives such as money, (grocery) gift cards, recipe books, exercise equipment [12, 31–34], reimbursements for travel or study expenses [11, 29, 34, 36, 37], and providing food and drinks during appointments [12, 33, 35] can significantly increase accessibility and attractiveness and are related to a greater retention of participants. Other successful examples offered include offering participants certificates of intervention completion, and/or marking the end of a health intervention research initiative with a ceremony or party.

## Discussion

This systematic review of reviews aimed to synthesize effective encouragement, facilitation, and retention strategies that researchers can implement to better reach, involve and maintain underserved populations in health intervention research. By integrating findings across 18 reviews spanning several health domains and underserved population subgroups, we identified sets of strategies that broadly and consistently serve these goals. Our synthesis shows that several strategies exist which often operate across multiple phases of the research process (e.g., simultaneously supporting recruitment, involvement, and/or retention). However, besides cross-domain and population subgroup consistencies and divergences, some persistent gaps also became apparent. Most notably, the lack of explicit attention to retention of underserved populations as a primary focus in health intervention research and the scarcity of evidence regarding the effectiveness of individual strategies.

Five encouragement strategies were identified (see also Table 3) that can support researchers in better reaching underserved populations in health intervention research: (1) engaging and interacting with the target population during community events, (2) stimulating the collaboration with communities around the target population and/or partnering with intermediaries such as healthcare providers, (3) tailoring materials, information, study design and communication to the target population, (4) building a trust relationship with the target population and their community and being respectful towards their needs, and (5) passive inclusion processes to increase the pool of potential participants from the target population. These strategies all target either end users or intermediary groups (e.g., healthcare providers) and stress that it is vital to directly engage and collaborate with these target groups.

These encouragement strategies—with exception of passive inclusion procedures—are not only focused on raising awareness about and promoting health intervention projects within underserved populations. That is, these strategies can also be used for relationship management between participants, intermediaries and researchers. Many of these strategies also support retention implicitly, for example by fostering trust, familiarity, and perceived relevance of the research. However, retention by itself was rarely a primary outcome in the included reviews.

Moreover, many strategies used as facilitation strategies (see also Table 3) by supporting researchers in recruiting underserved populations in health intervention research (e.g., accessibility and incentives) also support their retention over the longer term. Strategies such as contact flexibility, research location accessibility, transportation support, and incentives can both reduce practical barriers to participation as well as prevent drop‑out after enrollment. Return on investment opportunities, for example by offering participants (and/or healthcare providers) free services and (non-)monetary incentives, also support sustained engagement.

This systematic review of reviews had several strengths. First, this review focused on the understudied topic of reaching, involving, and maintaining underserved populations within health intervention research [10, 20, 24]. Second, this review examined strategies that targeted other stakeholders besides the end users themselves, that is, the intermediaries. Finally, the (systematic) review of reviews methodology allowed for a comprehensive overview of the available evidence regarding encouragement, facilitation, and retention strategies through the aggregation of the findings from multiple reviews across health domains and underserved population subgroups. This allowed us to summarize the most widely used strategies and identify literature gaps, such as the relative lack of focus on retention strategies in the literature. Taken together, our integrated synthesis sheds light on the current state of art and offers focal points for further advancement of equitable research practices. By situating recruitment and retention strategies in one framework and identifying areas with inconsistent or lacking evidence, we hope to guide researchers and policymakers who seek to improve the inclusion of underserved populations in (research on) health interventions.

While this review offered valuable insights, it is important to acknowledge several limitations that may hamper interpretation of findings. First, only a small number of the included reviews synthesized quantitative evidence, and only one review incorporated a meta-analysis. While qualitative studies provide rich, contextual insights, the lack of quantitative studies poses a challenge in establishing a clear link between the strategies and their effectiveness in recruitment and retention. Second, 4 of the 18 included reviews received a weak quality score [11, 24, 26, 36]. However, the quality of the reviews does not necessarily reflect the quality of the individual studies included in the review and/or the strategies included in the reviews. Third, due to the narrow focus of this review and the applied selection criteria, potentially relevant strategies may have been excluded. As a result, several approaches and frameworks from the broader literature were not captured in this review. A relevant example is highlighted by (community-based) participatory research and co-design [38, 39]. These methodological approaches offer valuable strategies in support of the meaningful involvement of (communities of) underserved populations in health intervention research. However, these strategies fell beyond the scope of the current review of reviews. That is, these strategies move beyond discrete recruitment and retention strategies and emphasize equitable partnership and cocreation with vulnerable populations to develop better fitting health interventions. Future syntheses with a broader scope on the inclusion of underserved populations in health intervention research should integrate these—and other—perspectives. That is, future syntheses that more fully describe the relational and structural mechanisms involved in the sustained engagement of underserved populations in health intervention research are needed. Building on this suggestion, another relevant example is highlighted by approaches related to data justice and health equity, which provide important additional perspectives on such mechanisms [42, 43]. These approaches address issues related to power imbalances, historical exclusions, fair data practices, and social determinants of health that influence participation. Again, although these perspectives enrich our understanding of community engagement, their breadth extends beyond our scope of discrete recruitment, involvement and retention strategies. Including also these perspectives in future syntheses could enrich the knowledge base on the structural and ethical dimensions of participant inclusion in health intervention research. Further examples from the broader literature that could not be integrated in the current review of reviews include good practices such as presenting research outcomes to the community and intermediaries to strengthen participants’ trust and engagement. Moreover, maintaining multiple forms of contact with participants, along with having contact information for a member of their immediate family, can be beneficial during follow-up moments. Also, the value of fostering a reciprocal relationship where researchers learn from the community, was not evident in the present review. Finally, this review did not address the challenges posed by the General Data Protection Regulation (GDPR), which, while ensuring privacy protection, can introduce administrative and practical barriers that hinder the recruitment of underserved populations, ultimately limiting their participation in research.

The inclusion of underserved populations in research is a critical issue in health intervention research. The findings of this systematic review of reviews imply that future research needs to focus more strongly on retention strategies. Specifically, future studies should include retention as a primary outcome rather than as an assumed secondary benefit of recruitment or facilitation strategies. Quantitative studies that examine the efficacy of recruitment and retention strategies (in terms of desired outcomes) and assess their generalizability to diverse contexts are also essential. Our cross-review synthesis also highlights potential priority directions for future research, such as the need for more rigorous evaluations of recruitment and retention strategies across contexts, improved reporting standards to enhance the comparability of findings, and the integration of structural and relational perspectives to clarify the mechanisms underlying the sustained engagement of underserved populations. Additionally, exploring alternative research designs to examine (combinations of) recruitment and retention strategies is necessary to enable meaningful comparisons. While it appears that some strategies can simultaneously aid in the recruitment and retention of underserved populations in research, it is essential to consider the context-specific potential of each. A multifaceted approach (i.e. recruitment intervention), consisting of multiple strategies as active components is recommended to address the diverse needs of underserved populations. Such an approach can highlight specific components that will enhance the understanding of how each individual component contributes to the overall goal. This also facilitates the replication and implementation of these strategies in different settings.

## Conclusions

This systematic review of reviews aimed to synthesize existing intervention strategies for improving the inclusion of underserved populations in health intervention research across domains and assess evidence on their effectiveness. The findings underscore the importance of a multifaceted recruitment approach, that incorporates different strategies to serve diverse goals throughout the whole intervention process. To effectively increase the involvement of underserved populations in health intervention research, researchers should prioritize early engagement of members of underserved populations during the development of health interventions. This will ensure that all materials and interaction methods are culturally, linguistically, and contextually tailored to their specific needs and values. Language, media, and communication channels that resonate with the community, fostering greater relevance and connection, are helpful to this end. Researchers are advised to collaborate closely with local organizations and healthcare providers to facilitate recruitment through leveraging existing networks. The availability of alternative participation options, such as community-based sessions, virtual platforms, and flexible schedules and locations, can make participation more accessible. Provision of incentives—both financial and non-financial—are encouraged to increase motivation and support retention of participants. Importantly, these efforts should be paired with a consistent commitment to building trust, through transparent communication, respecting cultural norms, ensuring confidentiality, and providing continuous support to participants throughout the research process. By adopting these approaches researchers can contribute to more inclusive and equitable health interventions, thereby reducing health disparities and improving outcomes for all communities.

## Supplementary Information

Below is the link to the electronic supplementary material.

**Supplementary Material 1**: **Supplementary Table S1**

## Data Availability

All data generated or analyzed during this study are included in this published article.

## Abbreviations

SES: Socioeconomic Status
PRISMA: Preferred Reporting Items for Systematic reviews and Meta-Analyses
HIV: Human Immunodeficiency Virus
GDPR: General Data Protection Regulation
