# Supplementary material for: Strategies to reach, involve, and maintain underserved populations in health intervention research: a systematic review of reviews
Source: Int J Equity Health. 2026 May 7;25:159. doi: 10.1186/s12939-026-02858-3 (PMC13321629; doi:10.1186/s12939-026-02858-3)
Supplement: Supplementary file 1 — Supplementary Material 1: Supplementary Table S1 [file 12939_2026_2858_MOESM1_ESM.docx]

**Supplementary Appendix**

**Table S1**

*Overview of excluded articles after full article review.*

| **Authors** |  | **Year** |  | **Title** |
| --- | --- | --- | --- | --- |
| ***Not health intervention research*** | | | | |
| Brockhoven, F., Raphael, M., Pashayan, N., & Arteaga, I. |  | 2025 |  | Increasing participation of underrepresented groups in cancer early detection research: a scoping review. |
| Nisha, M., Bateson, D., Saville, M., Nightingale, C. E., Bavor, C., Canfell, K., & Smith, M. A. |  | 2025 |  | The role of non-medical providers in increasing access to cervical screening: a scoping review. |
| Torres, J., & Tajima, E. |  | 2025 |  | Approaches to Filipino American Community Engagement in Prevention Research: A Scoping Review |
| Perowne, R., Rowe, S., Lajevardi, A., Bingham, L., Parry, E., Grey, G., Thomas, P. C., & Gutman, L. M. |  | 2025 |  | Barriers and Facilitators to The Involvement of Under-Represented Children and Young People (aged 8–25) in Mental Health Research – a Systematic Review |
| Assfaw, A. D., Babulal, G. M., Balls‐Berry, J., Mozersky, J., Moulder, K., & Morris, J. C. |  | 2025 |  | Community engagement, recruitment, and retention of minoritized participants in Alzheimer's disease and related dementia research: A systematic review of disparities |
| Peters CJ, Greve JM, Karbasi A, Walker M, Adesanya L |  | 2025 |  | Recruitment and retention strategies for improving representation in clinical research: A meta-synthesis |
| Törnävä, M., Palonen, M., Harju, E., Haapa, T., Rissanen, M. L., & Kylmä, J. |  | 2025 |  | Hard-To-Reach and Hidden Groups in Health-Related Research-A Scoping Review. |
| Lovell, H., Wicks, E., Thompson, H., Brace, L., Stacey, T., Rayment-Jones, H., & Harding, S. |  | 2025 |  | Recruitment, retention and reporting of ethnic representativeness in maternity trials: a scoping review |
| Brijnath, B., Croy, S., Sabates, J., Thodis, A., Ellis, S., de Crespigny, F., Moxey, A., Day, R., Dobson, A., Elliott, C., Etherington, C., Geronimo, M. A., Hlis, D., Lampit, A., Low, L. F., Straiton, N., & Temple, J. |  | 2022 |  | Including ethnic minorities in dementia research: Recommendations from a scoping review. |
| Bradway, M., Perestelo-Perez, L., Torres-Castaño, A., Wagner, A. M. C., Álvarez-Malé, M. L., Zamorano, G. B. Z., & Dyb, K. |  | 2025 |  | Making “inclusion” more than a buzzword: A critical interpretive synthesis of literature about recruiting seldom-heard groups in health research |
| Ibrahim S, Sidani S. |  | 2013 |  | Strategies to recruit minority persons: a systematic review |
| Gilmore-Bykovskyi AL, Jin Y, Gleason C, Flowers-Benton S, Block LM, Dilworth-Anderson P, Barnes LL, Shah MN, Zuelsdorff M. |  | 2019 |  | Recruitment and retention of underrepresented populations in Alzheimer's disease research: A systematic review |
| Wieland ML, Njeru JW, Alahdab F, Doubeni CA, Sia IG. |  | 2021 |  | Community-Engaged Approaches for Minority Recruitment Into Clinical Research: A Scoping Review of the Literature |
| Barros AB, Dias SF, Martins MR. |  | 2015 |  | Hard-to-reach populations of men who have sex with men and sex workers: a systematic review on sampling methods |
| Waheed W, Hughes-Morley A, Woodham A, Allen G, Bower P. |  | 2015 |  | Overcoming barriers to recruiting ethnic minorities to mental health research: a typology of recruitment strategies |
| Godbole N, Kwon SC, Beasley JM, Roberts T, Kranick J, Smilowitz J, Park A, Sherman SE, Trinh-Shevrin C, Chodosh J. |  | 2022 |  | Assessing Equitable Inclusion of Underrepresented Older Adults in Alzheimer's Disease, Related Cognitive Disorders, and Aging-Related Research: A Scoping Review |
| Wong R, Amano T, Lin SY, Zhou Y, Morrow-Howell N. |  | 2019 |  | Strategies for the Recruitment and Retention of Racial/Ethnic Minorities in Alzheimer Disease and Dementia Clinical Research |
| Liljas AEM, Walters K, Jovicic A, Iliffe S, Manthorpe J, Goodman C, Kharicha K. |  | 2017 |  | Strategies to improve engagement of 'hard to reach' older people in research on health promotion: a systematic review |
| Gonzalez M, Phoenix M, Saxena S, Cardoso R, Canac-Marquis M, Hales L, Putterman C, Shikako-Thomas K. |  | 2021 |  | Strategies used to engage hard-to-reach populations in childhood disability research: a scoping review |
| Goedhart NS, Pittens CACM, Tončinić S, Zuiderent-Jerak T, Dedding C, Broerse JEW. |  | 2021 |  | Engaging citizens living in vulnerable circumstances in research: a narrative review using a systematic search |
| Thakur N, Lovinsky-Desir S, Appell D, Bime C, Castro L, Celedón JC, Ferreira J, George M, Mageto Y, Mainous III AG, Pakhale S, Riekert KA, Roman J, Ruvalcaba E, Sharma S, Shete P, Wisnivesky JP, Holguin F. |  | 2021 |  | Enhancing Recruitment and Retention of Minority Populations for Clinical Research in Pulmonary, Critical Care, and Sleep Medicine: An Official American Thoracic Society Research Statement |
| Koyuncu A, Ishizumi A, Daniels D, Jalloh MF, Wallace AS, Prybylski D. |  | 2023 |  | The Use of Adaptive Sampling to Reach Disadvantaged Populations for Immunization Programs and Assessments: A Systematic Review |
| Sedrak MS, Freedman RA, Cohen HJ, Muss HB, Jatoi A, Klepin HD, Wildes TM, Le-Rademacher JG, Kimmick GG, Tew WP, George K, Padam S, Liu J, Wong AR, Lynch A, Djulbegovic B, Mohile SG, Dale W |  | 2020 |  | Older adult participation in cancer clinical trials: A systematic review of barriers and interventions |
| Helms YB, Hamdiui N, Kretzschmar MEE, Rocha LEC, van Steenbergen JE, Bengtsson L, Thorson A, Timen A, Stein ML. |  | 2021 |  | Applications and Recruitment Performance of Web-Based Respondent-Driven Sampling: Scoping Review |
| Bonevski B, Randell M, Paul C, Chapman K, Twyman L, Bryant J, Brozek I, Hughes C. |  | 2014 |  | Reaching the hard-to-reach: a systematic review of strategies for improving health and medical research with socially disadvantaged groups |
| Provencher V, Mortenson WB, Tanguay-Garneau L, Bélanger K, Dagenais M. |  | 2014 |  | Challenges and strategies pertaining to recruitment and retention of frail elderly in research studies: a systematic review |
| ***Does not report strategies for reaching, involving, or retaining underserved populations*** | | | | |
| Wagman, H., Iseyas, N., Mohmand, Z., Watson, M., Mahant, S., & Do, V. |  | 2025 |  | Methods for engaging vulnerable and marginalized children through community based participatory research: a scoping review. |
| Sam, N., Hill, J., & Hamer, O. |  | 2023 |  | Recruiting adults of ethnic minorities into clinical trials: a synthesis of strategies |
| Nisha Godbole, Simona C Kwon, Jeannette M Beasley, Timothy Roberts, Julie Kranick, Jessica Smilowitz, Agnes Park, Scott E Sherman, Chau Trinh-Shevrin, Joshua Chodosh |  | 2023 |  | Assessing Equitable Inclusion of Underrepresented Older Adults in Alzheimer’s Disease, Related Cognitive Disorders, and Aging-Related Research: A Scoping Review. |
| Iflaifel, M., Hall, C. L., Green, H. R., Willis, A., Rennick-Egglestone, S., Juszczak, E., Townsend, M., Martin, J., & Sprange, K. |  | 2024 |  | Strategies to improve recruitment in mental health clinical trials: a scoping review (RE-MIND study). |
| Hazlitt, R., Nguyen, K., Wilson, A., Clark, P., Ghebrehiwet, M., Autaubo, J., Turner, N., Anderson, B., Ford, A. I., & Vassar, M. |  | 2025 |  | Diversity, equity, and inclusion in asthma clinical trials: A cross-sectional analysis |
| Inam, E., Güney, S. & Roes, M. |  | 2025 |  | Recruitment strategies for Turkish immigrants in dementia care research: a scoping review |
| ***Not a review article*** | | | | |
| Smith, H., Khan, S., Wilson, A., Autaubo, J., Clark, P., Ghebrehiwet, M., Livingston, R., Cobbs, R., & Vassar, M. |  | 2024 |  | Recruitment and Retention Strategies for Historically Marginalized Populations in Colorectal Cancer Trials: A Cross-Sectional Analysis Using Systematic Review Methods |
| Pardhan, S., Sehmbi, T., Wijewickrama, R., Onumajuru, H., & Piyasena, M. P. |  | 2025 |  | Barriers and facilitators for engaging underrepresented ethnic minority populations in healthcare research: an umbrella review |
| Owusu-Addo, E., Bennor, D. M., Orkin, A. M., Chan, A. W., Welch, V. A., Treweek, S., Green, H., Feldman, P., Ghersi, D., Brijnath, B., & RECONSIDER Extension Group |  | 2024 |  | Recruitment, retention and reporting of variables related to ethnic diversity in randomised controlled trials: an umbrella review. |
| Hwang, D. A., Lee, A., Song, J. M., & Han, H. R. |  | 2021 |  | Recruitment and retention strategies among racial and ethnic minorities in web-based intervention trials: Retrospective qualitative analysis |
| ***Wrong study population*** | | | | |
| Ruth Mackenzie-Stewart, Cassandra de Lacy-Vawdon, Niamh Murphy, Ben J Smith |  | 2023 |  | Engaging adults in organized physical activity: a scoping review of recruitment strategies |
| Frew, P. M., Saint-Victor, D. S., Isaacs, M. B., Kim, S., Swamy, G. K., Sheffield, J. S., Edwards, K. M., Villafana, T., Kamagate, O., & Ault, K. |  | 2014 |  | Recruitment and retention of pregnant women into clinical research trials: An overview of challenges, facilitators, and best practices |
| ***Ineligible study design*** | | | | |
| Dabiri, S., Raman, R., Grooms, J., & Molina-Henry, D. |  | 2024 |  | Examining the Role of Community Engagement in Enhancing the Participation of Racial and Ethnic Minoritized Communities in Alzheimer's Disease Clinical Trials. |
| Helm, A., Kaufman, M., Glassy, N., Rodríguez, C. P., Shaffer, P., Nelson, B. B., & Smelson, D. |  | 2025 |  | Strategies for recruiting participants underrepresented in clinical research: A scoping review |
